# Efficient Optimization of Stimuli for Model-Based Design of Experiments to Resolve Dynamical Uncertainty

**DOI:** 10.1371/journal.pcbi.1004488

**Published:** 2015-09-17

**Authors:** Thembi Mdluli, Gregery T. Buzzard, Ann E. Rundell

**Affiliations:** 1 Weldon School of Biomedical Engineering, Purdue University, West Lafayette, Indiana, United States of America; 2 Mathematics Department, Purdue University, West Lafayette, Indiana, United States of America; University of California San Diego, UNITED STATES

## Abstract

This model-based design of experiments (MBDOE) method determines the input magnitudes of an experimental stimuli to apply and the associated measurements that should be taken to optimally constrain the uncertain dynamics of a biological system under study. The ideal global solution for this experiment design problem is generally computationally intractable because of parametric uncertainties in the mathematical model of the biological system. Others have addressed this issue by limiting the solution to a local estimate of the model parameters. Here we present an approach that is independent of the local parameter constraint. This approach is made computationally efficient and tractable by the use of: (1) sparse grid interpolation that approximates the biological system dynamics, (2) representative parameters that uniformly represent the data-consistent dynamical space, and (3) probability weights of the represented experimentally distinguishable dynamics. Our approach identifies data-consistent representative parameters using sparse grid interpolants, constructs the optimal input sequence from a greedy search, and defines the associated optimal measurements using a scenario tree. We explore the optimality of this MBDOE algorithm using a 3-dimensional Hes1 model and a 19-dimensional T-cell receptor model. The 19-dimensional T-cell model also demonstrates the MBDOE algorithm’s scalability to higher dimensions. In both cases, the dynamical uncertainty region that bounds the trajectories of the target system states were reduced by as much as 86% and 99% respectively after completing the designed experiments *in silico*. Our results suggest that for resolving dynamical uncertainty, the ability to design an input sequence paired with its associated measurements is particularly important when limited by the number of measurements.

## Author Summary

Many mathematical models that have been developed for biological systems are limited because the complex systems are not well understood, the parameters are not known, and available data is limited and noisy. On the other hand, experiments to support model development are limited in terms of costs and time, feasible inputs and feasible measurements. MBDOE combines the mathematical models with experiment design to strategically design optimal experiments to obtain data that will contribute to the understanding of the systems. Our approach extends current capabilities of existing MBDOE techniques to make them more useful for scientists to resolve the trajectories of the system under study. It identifies the optimal conditions for stimuli and measurements that yield the most information about the system given the practical limitations. Exploration of the input space is not a trivial extension to MBDOE methods used for determining optimal measurements due to the nonlinear nature of many biological system models. The exploration of the system dynamics elicited by different inputs requires a computationally efficient and tractable approach. Our approach plans optimal experiments to reduce dynamical uncertainty in the output of selected target states of the biological system.

## Introduction

Since experiments can be expensive and time consuming, it is important that they are planned to generate useful data. Traditional design of experiments is a well established field and has led to many advances in biology and medicine. The data obtained from strategically designed experiments has facilitated the creation of mathematical models that relate experimental stimuli to measurable outcomes. These models typically describe the system’s input-output relationship but fail to capture or encode knowledge of the system’s internal mechanisms and processes. Mechanistic and semi-mechanistic mathematical models encode the current understanding of the internal processes of the biological system even though many of these internal states or species are not directly measurable. These mechanistic models can be used to support optimal experiment design that considers the current knowledge of the system interactions and practical experimental constraints. In recent literature this type of experiment design has been referred to as model-based design of experiments (MBDOE). MBDOE produces experiments meant to reduce some measure of uncertainty in the associated model while respecting cost, time and resource constraints. Most MBDOE strategies can be categorized by three types of objectives: (1) reducing model parameter uncertainty [1–7], (2) discriminating among possible models [8–13], and (3) reducing dynamical uncertainty [14–17]. This work advances current abilities to design experiments to resolve the trajectories of target states of a biological system model, thereby reducing its dynamical uncertainty.

Many of the MBDOE strategies that support reduction of parameter uncertainty and model discrimination rely on linear approximations that are locally optimal to design an experiment by optimizing a criterion of the Fisher Information Matrix (FIM) [15, 18–21]. Such techniques use the local sensitivities of parameters to design an optimal experiment which requires an initial estimate of the unknown parameters. Most biological system models are not well characterized, as data is limited and noisy, so initial estimates of the model parameters are inaccurate. Furthermore, biological models are typically nonlinear, so a poor initial parameter estimate is likely to result in a local minimum, which may result in sub-optimal experiment design. Alternatively, the Sigma Point method refines estimates of the characteristic values of the parameter statistics [22, 23]. This method is computationally efficient and gives a better approximation of the covariance matrix when compared to the FIM-based and boot-strap methods. However, this method is not applicable when the biological system is not well characterized with existing experimental data. These local methods for MBDOE are limited to scenarios when initial estimates for the model parameters or their distributions are already fairly well known.

To overcome the shortcomings of local MBDOE strategies, global approaches that consider the entire uncertain parameter range have been developed. Global optimization has been used to improve the FIM-based design [24] and local deterministic optimization of the likelihood with multi-start initial parameter estimates [25] have been previously employed. Some Bayesian MBDOE methods use Monte Carlo generated estimates of the parameter confidence intervals to design a global optimal experiment [16, 26–30]. A comparison of the FIM-based and Bayesian-based MBDOE methods by Weber et al. [31] shows that Bayesian methods give more accurate and informative designs. However, all of these global methods are computationally prohibitive for large numbers of highly uncertain parameters and mathematical models that are computationally intensive. The limitation due to computation has been partially abrogated by methods that utilize surrogate models to approximate the mathematical model of the system. Herein, as in our previous work [14, 15], we use a sparse grid interpolation tool to approximate the biological model.

To find the optimal design a search has to be performed over all of the experimental factors that define the feasible experimental perturbations and potential measurements. A complete exploration of the full design space over the uncertain parameter space for an optimal experiment design is impractical due to the combinatorial explosion of possible experimental factors evaluated for all possible model parameterizations. To solve the problem, most MBDOE strategies constrain the design space to a subset of the possible experiments. This reduces the design space by assuming a small predefined set of possible measurement points, measurable species and/or input levels [9, 31–33]. Herein, we define the input to be the exogenous experimental factors, perturbations and/or stimuli that are applied to the biological system. Other designs have considered finding an optimal input sequence while the times and measurement species are defined *a priori* [2, 10, 24, 34]. To consider both optimal measurements and an optimal input sequence, some strategies [16, 35] evaluate the optimality of a pre-defined set of specified inputs and measurement points. All of these approaches may result in sub-optimal experiment designs if the best experiment is not among the specified experimental options.

Ideally, experiment design determines the optimal solution within a feasible space defined by all the possible experimental measurements and inputs. Since different inputs can elicit dramatically different dynamics for some nonlinear systems, evaluating experiments for optimal inputs is not necessarily a trivial extension of the global techniques for identifying optimal measurements. Herein, we describe and evaluate a computationally efficient and tractable method for performing global MBDOE to define optimal experimental inputs and measurements to reduce the uncertainty in continuous nonlinear system dynamics. The method utilizes the sparse grid surrogate model technique first employed for global experimental designs presented in [14, 15], which selected only optimal measurements and not optimal inputs. We proposed an earlier version [36] of our input design MBDOE algorithm that used a combination of scenario trees and sparse grid interpolation to screen the input design space for optimal inputs that will generate diverse dynamics of the uncertain nonlinear system. This paper is an extension of that work. Herein we utilize a greedy search to determine the input magnitude to be applied using a method that is similar to that in [37] which determined near-optimal measurements in polynomial time. Our MBDOE algorithm is made even more computationally efficient and robust than our previous sparse grid based strategies due to the uniform sampling over the dynamical space via careful selection of representative parameters, the optimization criteria for optimal experiment design for a target system, and the repeated update of probability weights over the dynamical space using predicted data. Our new MBDOE method develops a strategic experiment design that yields highly informative data that can be used to constrain the system dynamics to dynamical uncertainty regions.

In the methods section, we describe the experiment design problem and define the methods used to solve the problem as sequential optimizations to first select an optimal input sequence and, subsequently, specify the associated optimal measurement pairs (species and time points). In the results section, we explore the optimality of the derived experiment design solution using a small 3-dimensional Hes1 oscillatory model and demonstrate the scalability of this MBDOE strategy with a 19-dimensional T-cell receptor model. The global applicability, support of hybrid experiment designs, and limitations of this MBDOE approach are discussed to conclude the paper.

## Materials and Methods

### Dynamical Model Definition

This MBDOE algorithm is designed to resolve dynamical uncertainties associated with the biological system by reducing the predicted variance in the system dynamics. The system is described by nonlinear ordinary differential equations of the general form:

$$\begin{array}{c} \dot{x}=f\left(x,u,\theta ,\theta^{{}^{\prime }},t\right),\;\;\;\;x\left(t_{0}\right)=x_{0} \end{array}$$ (1)

where **f** is a deterministic smooth function of $\text{x}\in {\mathbb{R}}^{n_{x}}$, the states of the system, $\theta \in {\mathbb{R}}^{n_{p}}$, the known model parameters, $\theta^{\prime }\in {\mathbb{R}}^{n_{p^{\prime }}}$, the unknown model parameters, and $u\in {\mathbb{R}}^{n_{u}}$ the exogenous inputs. Using control vector parameterization (CVP) [38], we represent **u** = [***u***(*τ*_1_), …, ***u***(*τ*_*N*_)] ∈ ℝ^*n*_*u*_∗*N*^, as vector-valued function of the system inputs that can change value at each discrete time point, *τ*_*j*_, where *j* = 1, …, *N*. There is uncertainty in the dynamics of the system which is generated by the uncertainty in the values of the unknown model parameters, ***θ***′ ∈ Ω where Ω is a compact set.

It is common in biological systems that not all states of this system are experimentally measurable. All feasible measurements for this system are modeled by:

$$\begin{array}{c} y=h(x,u,\theta ,\theta^{{}^{\prime }},t), \end{array}$$ (2)

where **h** is a smooth vector-valued function that relates to the system’s internal states described in Eq (1) and $\text{y}\in {\mathbb{R}}^{n_{m}}$. We abbreviate Eq (2) by the notation **y**(**u**, **θ**′, *t*). The user defines the number of measurements that they would like to have designed, *K*, as well as the set of possible times for measurement, 𝕋, by either specifying discrete time points or defining a time resolution, *δt*, between *T*_*I*_ and *T*_*F*_, the initial and final model simulation times, respectively.

Our MBDOE algorithm designs experiments that reduce dynamical uncertainty in a target system. This target system is a subset of the system states termed, target states, **x**_***T***_, when stimulated by a target input, **u**_***T***_. This approach employs the concept of a target system also used to evaluate an alternative MBDOE strategy [16].

We consider dynamical uncertainty in the measurable states, **y**, and the target states, **x**_***T***_, generated by the uncertainty in the unknown parameters, ***θ***′. As a result of limited and noisy data, often biological system models will fit the data with a wide range of parameters values and associated dynamics. Therefore, for an optimal design, we need to consider all the possible data-consistent dynamical scenarios which are a function of the parameter space, Ω.

### Optimization Problem Definition

The optimal experiment, D* ∈ 𝔻, defines the optimal input stimulation and optimal measurement pairs that resolve the target state dynamics. This design is defined by a piecewise input sequence, u* (Eq (3)), and associated measurements M* = {(*m*_*k*_, *t*_*k*_):*k* = 1,2,…*K*} ∈ 𝕄, which defines *K* measurement pairs by the index of a measurement species *m*_*k*_ and its corresponding measurement time *t*_*k*_.

$$\begin{array}{c} u^{*}=\{\begin{array}{cc} u_{1}^{*} & \tau_{1}\leq t\leq \tau_{2}\\ \vdots & \vdots \\ u_{j}^{*} & \tau_{j}\leq t\leq \tau_{j+1}\\ \vdots & \vdots \\ u_{N}^{*} & \tau_{N}\leq t\leq T_{F} \end{array} \end{array}$$ (3)

We choose D* as the experimental design that maximizes a measure of information gained from an experiment

$$\begin{array}{c} D^{*}=\underset{u\in {\mathbb{R}}^{n_{u}*N},M\in 𝕄}{\mathrm{argmin}}\gamma \left(u\right). \end{array}$$ (4)

The target state dynamical uncertainty (TDU), *γ*(**u**), is quantified by the sum of the maximum variance in each target state that results when analyzed assuming the input sequence, **u**, has been applied and measurements, *M*, exist to constrain the uncertain parameter space:

$$\begin{array}{c} \gamma \left(u\right)=\sum_{i}\max\left(\text{Var}\left(x_{Ti}\left(t\right)\right)|\left(u,M\right)\right) \end{array}$$ (5)

where the maximum variance of the *i* ^*th*^ target state, max(Var(*x*_**T***i*_(*t*)∣(**u**, *M*)), is chosen across the simulated time *t*, from the initial, *T*_*I*_ to the final time, *T*_*F*_.

The ideal solution for the above optimization problem simultaneously solves for the optimal input vector, u*, together with the optimal measurements, M*. This is generally a computationally intractable problem due to the combinatorial explosion of all possible selections for the experiment design complicated by the expense of evaluating the design with the model for all possible uncertain parameter values. Our approach proposes a computationally efficient method to approximate both the optimal input vector and associated optimal measurement points by breaking the problem into smaller computationally feasible optimization problems. The first optimization problem solves for the input vector using a greedy method by minimizing the value of TDU at each iteration assuming a single best measurement is taken:

$$\begin{array}{c} u^{*}=\underset{u_{j}\in {\mathbb{R}}^{n_{u}}}{\mathrm{argmin}}\gamma \left(u\right),j=1,\cdots N. \end{array}$$ (6)

The optimal input vector is then used in the second optimization to determine the multiple optimal measurements points. This combined solution of input and measurements approximates the optimal experiment design defined in Eq (4).

**Fig 1:**
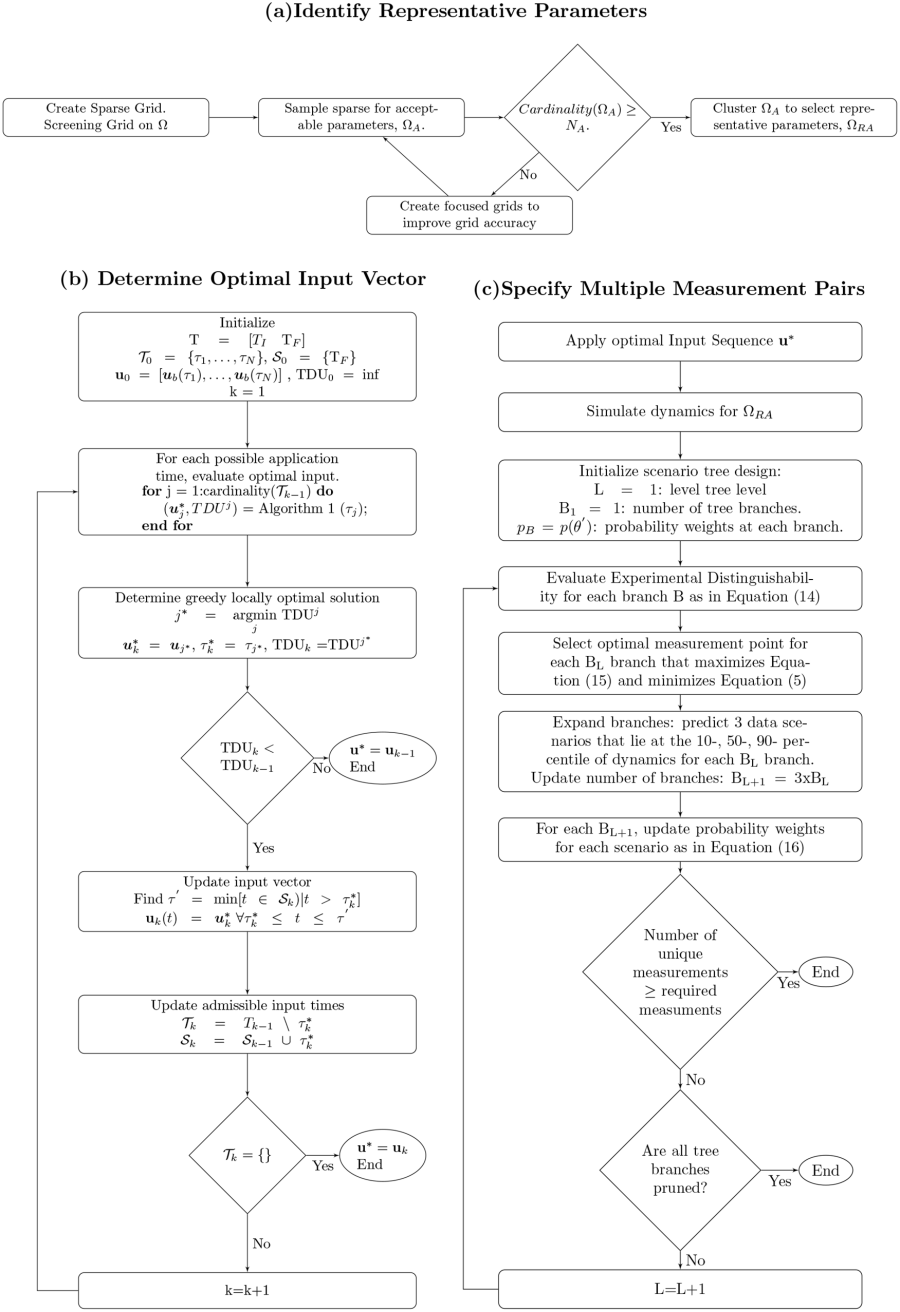
Flow charts describing the three steps for the MBDOE method. These describe (a) how acceptable and representative parameters are identified, (b) the process for determining the optimal input vector, and (c) the selection of multiple measurement pairs associated with the optimal input vector.

A flow-chart of the MBDOE algorithm is given in Fig 1 to display the sequence of events in determining the optimal experiment design: (a) identify representative parameters that maintain the diversity of simulated dynamics that fit the existing data, (b) determine the optimal input vector that minimizes TDU, and (c) specify associated multiple measurement pairs given an optimal input vector.

### Identify Representative Parameters

The first step of this process screens the uncertain parameter space, Ω, to identify the space of acceptable parameters, Ω_*A*_. The acceptable parameters are those that support model simulations to fit the available data as:

$$\begin{array}{c} \Omega_{A}=\{\theta^{{}^{\prime }}\in \Omega |\log_{10}(1+\sum_{\theta^{{}^{\prime }}\in \Omega }{\left(\frac{{\tilde{y}}_{i}\left(u,\theta^{{}^{\prime }},t\right)-{\hat{y}}_{i}\left(u,t\right)}{\sigma_{i}\left(t\right)}\right)}^{2})\leq T_{A}\} \end{array}$$ (7)

where ${\hat{y}}_{i}(\text{u},t)$ is the mean of the experimental data for the *i* ^*th*^ model output at the time point, *t*, collected with the applied input **u**, ${\tilde{y}}_{i}(\text{u},\theta^{\prime },t)$ is the corresponding model dynamics simulated with a parameter set, ***θ***′, and *σ*_*i*_(*t*) is the standard deviation of the data. In this work, we approximate ${\tilde{y}}_{i}(\text{u},\theta^{\prime },t)$ using a sparse grid interpolation tool over the uncertain space Ω for computational efficiency. *T*_*A*_ is the threshold for acceptability. This weighted least squares function has been used before to define acceptable parameters in [14, 15]. The parameter space, Ω, is sampled using Latin Hypercube Sampling (LHS). If the initial screen produces fewer acceptable parameters than *N*_*A*_, the desired number of acceptable parameter vectors that will support the experiment design, focused grids are created [15] to improve the resolution of the grid interpolant in the acceptable regions of the uncertain parameter space. LHS is also used to sample the focused grids to find more acceptable parameters until the *cardinality*(Ω_*A*_) ≥ *N*_*A*_. In this work, we define the *dynamical uncertainty region* to be the region spanned by the most extreme trajectories of the acceptable parameters.

#### Selection of Representative Parameters

We select representative parameters from the acceptable parameter space, Ω_*RA*_ ⊂ Ω_*A*_, that span the dynamics space, denoted by 𝕯_*A*_, simulated by the model over the acceptable parameter space. Our selection of Ω_*RA*_ effectively samples the parameters consistent with the number of experimentally distinguishable dynamics for robustness. We cluster the trajectories, *η* ∈ 𝕯_*A*_, where *η* = *φ*(*θ*′) is the trajectory associated with *θ*′, so that parameters that generate similar dynamics are grouped together. A spectral clustering method is used that employs eigenvalue analysis to estimate the number of clusters. The cluster number is determined at the largest gap of the eigenvalues of the normalized Laplacian matrix that is used in spectral clustering as in [39]. After the clusters are created, we select representative parameters from each cluster to form Ω_*RA*_. To preserve dynamical diversity, the representative parameters are sampled from the convex hull of each cluster using the MATLAB function *convhulln*. The number of representatives sampled from the convex hull of each cluster is equal to a user-specified number, **C**, with minimum sample size of three to ensure triangulation. Thus the representative parameters discretize the acceptable uncertain space into *N*_*RA*_ = **C**⋅(# of clusters) parameters that preserve the dynamical diversity. We use these representative parameters as an indirect means to approximate a uniform sampling from the acceptable trajectory space as described next.

#### Computational Efficiency Afforded by Dynamical Representative Parameters

For computational efficiency, our goal is to estimate the mean and variance of possible trajectories with uniform sampling in dynamical space. To be more precise, let ℓ_Ω_(*θ*′) be the likelihood of *θ*′ based on existing experimental data. Then one way to define the probability of *θ*′ is:

$$\begin{array}{c} p_{\Omega }\left(\theta^{{}^{\prime }}\right)=\frac{\ell_{\Omega }\left(\theta^{{}^{\prime }}\right)}{\sum_{\lambda \in \Omega_{A}}\ell_{\Omega }\left(\lambda \right)}. \end{array}$$ (8)

However, this focuses on the parameter space and may overweigh some regions of the dynamical space if the dynamics are relatively insensitive to changes in the parameter values. This is due to the fact that we obtained Ω_*A*_ by LHS on the parameter space. Instead, we want to sample uniformly in the dynamical space. Let 𝕯_*RA*_ = *φ*(Ω_*RA*_) be the set of trajectories simulated with the set of representative parameters. So the probability of a given trajectory, *η*, can be defined by:

$$\begin{array}{c} p_{D_{RA}}\left(\eta \right)=\frac{\ell_{D}\left(\eta \right)}{\sum_{\nu \in D_{RA}}\ell_{D}\left(\nu \right)}. \end{array}$$ (9)

This can be used to translate to a *p*_Ω_*RA*__(*θ*′) where *θ*′ generates a trajectory, *η*, that is contained in 𝕯_*RA*_. (Recall, *η* ∈ 𝕯_*A*_.) Thus, an alternative way to define the probability of *θ*′ over the experimentally distinguishable dynamical space is:

$$\begin{array}{c} p_{\Omega_{RA}}\left(\theta^{{}^{\prime }}\right)=\frac{\ell_{\Omega }\left(\theta^{{}^{\prime }}\right)}{\sum_{\beta \in \Omega_{RA}}\ell_{D}\left(\beta \right)}. \end{array}$$ (10)

Since 𝕯_*RA*_ spans the dynamical set represented within 𝕯_*A*_, its reduced membership is compensated for by these probabilities.

The representative acceptable parameter set and its associated probability weights enable us to estimate the expected values and variances for the measured and target states over the experimentally distinguishable dynamics as:

$$\begin{array}{c} \sum_{D_{A}}\left(f\right)\approx \sum_{\eta \in D_{RA}}p_{D_{RA}}\left(\eta \right)f\left(\eta \right)=\sum_{\theta^{{}^{\prime }}\in \Omega_{RA}}p_{\Omega_{RA}}\left(\theta^{{}^{\prime }}\right)f\left(\theta^{{}^{\prime }}\right). \end{array}$$ (11)

Table 1 specifies where this relationship in Eq (11) is used in the following equations to estimate the expected values and variances in a computationally efficient manner using the representative parameters.

**Table 1:** Mapping of functions to equation numbers to efficiently estimate necessary expected values and variances.

Equation number	Expected Value Description	f(θ′)
(14)	measurement variance, Var(*y*_*i*_(**u**, ⋅, *t*))	(*y*_*i*_(**u**,**θ**′, *t*)−*μ*_*i*_(*t*))^2^
(14) and (18)	expected experimental noise*, *E*(*σ*_*i*_(**u**, *t*))^2^	$\zeta_{b}+\zeta_{s}|y_{i}(\text{u},\theta^{\prime },t)|+\zeta_{t}|{\dot{y}}_{i}(\text{u},\theta^{\prime },t)|$
(5) and (18)	mean value, *μ*	*y*_*i*_(**u**, ***θ***′, *t*_*k*_)
(5)	target state variance, Var(*x*_**T***i*_(*t*))	(*x*_*Ti*_(**u**_*T*_, ***θ***′, *t*)−*μ*_*Ti*_(*t*))^2^

*the constants *ζ*_*b*_, *ζ*_*s*_, *ζ*_*t*_ are described in [12].

### Determine Optimal Input Vector

We propose solving the input vector using a greedy search algorithm. The input is discretized as in control vector parameterization (CVP) by the potential admissible input times 𝓣 = {*τ*_1_⋯*τ*_*N*_} and by potential magnitude levels. The number of possible input magnitudes is determined by the inputs bound [*u*_*min*_, *u*_*max*_] and resolution, *δu*:

$$\begin{array}{c} N_{u}=\frac{u_{max}-u_{min}}{\delta u}+1. \end{array}$$ (12)

The algorithm for the greedy search method to select the optimal input vector is detailed in the flowchart in Fig 1(b). The input magnitude is initially set to a base input level, **u**_1_ = ***u***_*b*_(*t*) ∀*t* ∈ [*T*_*I*_ *T*_*F*_], which is determined by the user. At each iteration, *k*, of the greedy search method, optimized input levels, $u_{j}^{*}$, for the admissible times, *τ*_*j*_ ∈ 𝓣_*k*−1_, are selected from all *N*_*u*_ possible admissible input magnitudes as described in Algorithm 1. We evaluate TDU^*j*^ for each of the admissible input times *τ*_*j*_ ∈ 𝓣_*k*−1_ and associated $u_{j}^{*}$ and select the input conditions, $\left(\tau_{j}^{*},u_{j}^{*}\right)$, that minimize the value of TDU according to Eq (6) as optimal. These optimal input conditions update the current input vector as follows:

$$\begin{array}{c} u_{k}^{*}=\{\begin{array}{cc} u_{j}^{*} & \tau_{j}^{*}\leq t\leq \tau^{{}^{\prime }}\\ u_{k-1}^{*} & \text{Elsewhere} \end{array} \end{array}$$ (13)

where *τ*′ is an input admissible time that has previously been optimized to update the vector, u*_k−1_. We define 𝓢_*k*−1_, a set of all previously optimized input admissible times such that $\tau^{\prime }=min[t\in {𝓢}_{k-1}|t>\tau_{j}^{*}]$. When the input vector is updated with optimal input conditions, $\left(\tau_{j}^{*},u_{j}^{*}\right)$, the input time, $\tau_{j}^{*}$ is moved from the set 𝓣_*k*−1_ to the set 𝓢_*k*−1_. This process continues iteratively, and the input vector is updated until 𝓣 is empty or no input magnitude change improves the value of TDU_*k*−1_ as shown in Fig 1(b).

**Algorithm 1** Algorithm for Selecting $u^{{*}_{j}}$

**Input:** *τ*_*j*_, ***u***_*min*_,***u***_*max*_, *δ* ***u***, 𝓢_*k*−1_, 𝓣_*k*−1_, u*_k−1_

**Output:** $u_{j}^{*}$ and corresponding TDU^*j*^

**for** p = 1:*N*_*u*_ **do** (Note: *N*_*u*_ = number of possible input magnitudes)

  1. Define $u_{j}^{p}=u_{min}+(p-1)\delta u$

  2. Simulate dynamics with **u** = ***u***_*p*_ for *τ*_*j*_ ≤ *t* < *τ*′ where *τ*′ = *min*[*t* ∈ 𝓢_*k*−1_∣*t* > *τ*_*j*_]

  3. Determine an associated measurement pair: *m*_*k*_ ∈ *n*_*m*_ and *t*_*k*_ ∈ 𝕋. *See* Eq (15)

  4. Update uncertain parameter probabilities given data for (*m*_*k*_, *t*_*k*_). *See* Eq (16)

  5. Estimate variance of target states, **x**_**T**_, with target input, **u**_**T**_, using updated uncertain parameter

  probabilities

  6. Calculate TDU^*p*^. *See* Eq (5)

**end for**

$p^{*}=\underset{p\in \{1,\cdots ,N_{u}\}}{\text{argmin}}$ TDU^*p*^. *See* Eq (6)

$u_{j}^{*}=u_{j}^{p^{*}}$

TDU^*j*^ = TDU^p*^

#### Determine an Associated Measurement Pair

Given an input vector, **u** ∈ ℝ^*n*_*u*_∗*N*^, a measurement pair is selected from the feasible measurement space defined by the $m_{k}^{th}$ entry in the **y** vector at a specific sample time point, *t*_*k*_. This pair is selected as the one that maximizes a Hampel smoothed distinguishability metric. We smooth the function of the distinguishability metric, *ξ*(*t*), over time using a Hampel identifier, Γ_*Hampel*_(•), to remove outliers and smooth the grid approximation (when used). The distinguishability metric quantifies the ability of an experiment to resolve the dynamics and is defined as:

$$\begin{array}{c} \xi_{i}\left(t\right)=\Gamma_{Hampel}(\frac{\text{Var}(y_{i}\left(u,\cdot ,t\right))}{E{\left(\sigma_{i}\left(u,t\right)\right)}^{2}}) \end{array}$$ (14)

where *ξ*_*i*_(*t*) is the output’s distinguishability metric for the for the *i* ^*th*^ measurable model output at each possible measurement time point. *E*(*σ*_*i*_(**u**, *t*))^2^ reflects the expected experimental noise of a measurement. If the experimental measurement noise is not available prior to running the experiments, we estimate the noise with an error model adopted from [14] as shown in Table 1. The optimal measurement pair satisfies:

$$\begin{array}{c} \left(m_{k},t_{k}\right)=\underset{i\in n_{m},t\in 𝕋}{\mathrm{argmax}}\xi_{i}\left(t\right). \end{array}$$ (15)

where *m*_*k*_ is the *i* ^*th*^ species of **y** and *t*_*k*_ is the sample time point.

This distinguishability metric is similar to the one introduced in [13] with the exception of the Hampel smoothing and the probability weights which were added to smooth the approximations for smaller numbers of sampled uncertain parameters as described in Eqs (8) to (11). The key point is that we focus on selecting measurements to distinguish dynamics rather than parameters. Hence, probability weights, *p*(*θ*′), are induced from a probability on dynamics in a way that will allow us to sample uniformly in dynamical space. This enables us to calculate the expected values and variances in Eq (14) over the experimentally distinguishable dynamics in a computationally efficient manner.

#### Update Uncertain Parameter Probabilities

Given the optimal measurement pairs (*m*_*k*_, *t*_*k*_), we use data that corresponds to the measurement point to assign and update parameter probability weights. Each of the uncertain parameters, ***θ***′ ∈ Ω_*RA*_, is assigned a probability weight to reflect the confidence given the simulation fitness for various scenarios. Herein, the priors of these probability weights are initially assigned uniformly across ***θ***′ ∈ Ω_*RA*_ and updated following Bayes theorem:

$$\begin{array}{c} p\left(\theta^{{}^{\prime }}|{\hat{y}}_{i}\left(u,t_{k}\right)\right)=\frac{p\left({\hat{y}}_{i}\left(u,t_{k}\right)|\theta^{{}^{\prime }}\right)p\left(\theta^{{}^{\prime }}\right)}{\tilde{p}\left({\hat{y}}_{i}\left(u,t_{k}\right)\right)}. \end{array}$$ (16)

$$\begin{array}{c} \tilde{p}\left({\hat{y}}_{i}\left(u,t_{k}\right)\right)\approx \sum_{\theta^{{}^{\prime }}\in \Omega_{RA}}p\left({\hat{y}}_{i}\left(u,t_{k}\right)|\theta^{{}^{\prime }}\right)p\left(\theta^{{}^{\prime }}\right). \end{array}$$ (17)

The posterior parameter probability, $p(\theta^{\prime }|{\hat{y}}_{i}(\text{u},t_{k}))$, under the observation is given by the prior probability, *p*(***θ***′), and the likelihood function, $p({\hat{y}}_{i}(\text{u},t_{k})|\theta^{\prime })$, normalized by the normalizing constant, $\tilde{p}({\hat{y}}_{i}(\text{u},t_{k}))$ which is approximated in Eq (17). ${\hat{y}}_{i}(\text{u},t_{k})$ is the data of the *i* ^*th*^ measurement species at the specific time point *t*_*k*_ collected under the application of the input **u**. If experimental data is available, then ${\hat{y}}_{i}(\text{u},t_{k})$ is the observed data. However, for our parallel design, we make predictions of the data for the selected measurement point since data is not available until the experiment design is over. In this case, ${\hat{y}}_{i}(\text{u},t_{k})$ is the prediction of the data for the selected species at the particular time point. With the design of multiple measurements employed in this method, data is predicted with a scenario tree design as described below and the likelihood of the predicted data is assumed to follow a Gaussian distribution:

$$\begin{array}{c} p({\hat{y}}_{i}\left(u,t_{k}\right)|\theta^{{}^{\prime }})=\frac{1}{\sqrt{2\pi E{\left(\sigma_{i}\left(u,t_{k}\right)\right)}^{2}}}\exp(-\frac{{\left({\hat{y}}_{i}\left(u,t_{k}\right)-y_{i}\left(u,\theta^{{}^{\prime }},t_{k}\right)\right)}^{2}}{2E{\left(\sigma_{i}\left(u,t_{k}\right)\right)}^{2}}) \end{array}$$ (18)

where *y*_*i*_(**u**, ***θ***′, *t*_*k*_)) is the model simulation of the dynamics of the model at the selected measurement point (*m*_*k*_, *t*_*k*_) with the parameter ***θ***′ under the input **u**.

### Specifying Multiple Measurement Pairs

Our design can identify multiple informative measurements given an optimal selected input vector, u*, as shown in Fig 1(c). We use an adaptation of the measurement scenario tree method [15] which makes multiple predictions of the data value from a given measurement point to allow the next informative point to be selected. In this work, we introduce the use of prior/posterior probability updates to minimize the bias due to outliers. The use of probabilities also enables the algorithm to work well with a smaller number of sampled uncertain parameters than previously, contributing to the computational efficiency. As in [15], a measurement scenario tree is initialized by a node which defines the first optimal measurement point. At each node, we determine the optimal sampling time point according to Eq (15) to maximize the distinguishability metric for each measurable species. Our MBDOE algorithm selects the measurement pair that minimizes the TDU according to Eq (5) as the optimal measurement pair for that node. Subsequent measurements (nodes) are chosen by considering three different scenarios (branches) from the node. These scenarios arise by predicting three possible outcomes for the measurement pair: previously these were defined by the minimum, mean, and maximum predicted measurement values. Herein, the cumulative distribution function is computed from the priors to span from the minimum to maximum predicted data values for that measurement pair. The three predicted possible outcomes are the simulated values at the measurement pairs that correspond to the 10^*th*^,50^*th*^, and 90^*th*^ percentiles. Using each of these predicted possible outcomes to approximate the data, ${\hat{y}}_{i}(\text{u},t_{k})$, the posterior for each branch are updated for the ***θ***′ ∈ Ω_*RA*_ as in Eq (16). Given the updated branch specific priors, the measurement pair specifying the next node in each branch is determined by the optimal measurement pair selected to minimize Eq (5) assuming the estimated posterior is the prior. This process is continued until the tree structure contains at least the specified number of unique measurements. The minimum number of levels in the tree is *log*_3_(*K*)+1 where *K* is the user-specified desired number of measurements.

### Computational Efficiency Afforded by Sparse Grid

Evaluating all possible experiments over the design space, 𝔻, using model simulations for a large uncertain parameter space, Ω, is computationally prohibitive. The sparse grid tool offers an efficient way to approximate the system’s output dynamics using interpolating polynomials by sampling an uncertain space in a systematic way to create a surrogate model for the system. The grid approximation is then used to interpolate additional points on the uncertain space without the cost of directly simulating the model.

#### Sparse grid Interpolation over Uncertain Parameter Space

A sparse grid approximation of the dynamics of the measured states over the uncertain parameter space is created using the process described previously [15]. With the sparse grid interpolant, points are sampled from Ω using LHS and their corresponding model output are approximated by:

$$\begin{array}{c} {\tilde{y}}_{i}\left(\cdot ,\theta^{{}^{\prime }},t\right)=L_{d,q}\left(t\right)\times y_{i}\left(\cdot ,\theta^{{}^{\prime }},T_{s}^{i}\right), \end{array}$$ (19)

where ${\tilde{y}}_{i}(\cdot ,\theta^{\prime },t)$ is the estimated value at time t for the *i* ^*th*^ model output and ${\text{y}}_{i}({\cdot ,\text{θ}}^{\prime },{\text{T}}_{s}^{i})$ is a vector of the *i* ^*th*^ model output sampled at time points, ${\text{T}}_{s}^{i}$, specified by the time-interpolating vector for the uncertain parameters, ***θ***′. Herein, the *L*_*d*, *q*_ are Lagrange polynomials where the degree *d* can be increased to improve the accuracy of the interpolant approximation while ensuring computational efficiency and q is used as the index to builds the polynomial, 0 < *q* < *d*.

#### Sparse grid Interpolation over Input Space

If the number of all possible input combinations exceed the maximum number of grid nodes predicted to create the sparse grid interpolation, the sparse grid interpolation tool is used to approximate the model dynamics over the input space. The sparse grid interpolant is sampled at the *N*_*u*_ possible input magnitudes and the corresponding model outputs are approximated for each ***θ***′ ∈ Ω_*RA*_ by:

$$\begin{array}{c} {\tilde{y}}_{i}\left(u,\cdot ,t\right)=L_{d,q}\left(t\right)\times y_{i}\left(u,\cdot ,T_{s}^{i}\right), \end{array}$$ (20)

The interpolated dynamics are used to characterize the experimental distinguishability metric (Eq (14)) for each of the *N*_*u*_ possible input magnitudes.

### Computational Implementation Details

The algorithm was coded in MATLAB (2013a). The Sparse Grid toolbox (v5.1.1) was obtained from http://www.ians.uni-stuttgart.de/spinterp [40] and integrated with our model-based experiment design algorithm. We minimize the error due to inaccurate interpolants by specifying the termination conditions based on their depth or by the relative or absolute errors of 10% and 5 respectively. The max depth is set to 3 and 5 for the parameter screening and focused grids, respectively, and 5 for the input grid. We set the data-consistent acceptability threshold *T*_*A*_ = 2, which correspond to two standard deviations. The Hes1 and T-cell receptor models for the results were numerically integrated using the stiff ode solver ode15s with the default settings. To increase computational efficiency, the sparse grid code and the mathematical models were vectorized. The code was run on four 8-core Intel Xeon 3.4 GHz CPUs each with 16 GB of memory and running the Windows 2003 server platform with the MATLAB Parallel Computing Toolbox. Our input design MBDOE algorithm code is available upon request from the corresponding author.

An upper bound on the number of model simulations used by our MBDOE algorithm can be approximated by:

$$\begin{array}{c} T_{eval}=\underset{\left(a\right)}{\underset{︸}{SG_{\theta }+N_{A}}}+\underset{\left(b\right)}{\underset{︸}{min\left(N_{u},SG_{u}\right)\times N_{RA}\times \frac{N(N+1)}{2}}}+\underset{\left(c\right)}{\underset{︸}{N_{RA}}} \end{array}$$ (21)

where *SG*_*θ*_ and *SG*_*u*_ are the number of model simulations (nodes) used to create sparse grid interpolants over the uncertain parameter and input space, respectively. An upper bound estimate of these values can be calculated using a Sparse Grid toolbox function, *spdim(maxDepth, Dim)* assuming the maximum possible depth and the dimension of the uncertain parameter or input space. The number of simulations required to build an accurate interpolant may be quite a bit lower than this approximation when the model is smooth over the uncertain parameter and/or input space. The terms of this upper bound approximation are mapped to the steps of Fig 1. The (a) term of Eq (21) is the total number of model simulations used to identify the representative parameters. The second term, (b), is the number of simulations that determine the optimal input vector. For this approximation, the sparse interpolant on the input space is only created if the estimated maximum number of nodes, *SG*_*u*_ required is less than *N*_*u*_. Furthermore, we assume a worst case scenario where the input magnitude changes at all *N* admissible times resulting in the fraction, $\frac{N(N+1)}{2}$. The third term, (c), is the number of model simulations used create the scenario tree to determine the measurement pairs.

## Results

### 3-Dimensional Problem: The Hes1 Model

To illustrate the effectiveness of our MBDOE strategy to resolve dynamical uncertainty of a target system, we use a simple Hes1 oscillator toy model [41]. This example demonstrates how an optimal perturbation enhances the ability of the MBDOE to reduce the dynamical uncertainty. The Hes1 model was previously used to demonstrate a Bayesian design of experiments strategy [16]. This model describes the changes in the level of the Hes1 transcription factor that is important in somitogenesis. It is modeled with an ODE system that describes the changes of the levels of the Hes1 mRNA, *m*, the cytosolic protein, *P*_1_, and the nuclear protein, *P*_2_, as shown:

$$\begin{array}{ccc} \frac{dm}{dt} & = & -km+\frac{1}{1+{\left(\frac{P_{2}}{P_{0}}\right)}^{h}},\\ \frac{dP_{1}}{dt} & = & -kP_{1}+\nu m-k_{1}P_{1},\\ \frac{dP_{2}}{dt} & = & -kP_{2}+k_{1}P_{1}. \end{array}$$ (22)

A description of the parameters of the model and their nominal values are listed in Table 2. The degradation rate, *k*, and protein transport rate, *k*_1_, are assumed to be known and their values are set to the nominal value while the rest of the parameters are considered unknown. The uncertain parameter space, Ω, is initially set to have bounds of 0.1 and 10 times previously reported nominal parameter values.

**Table 2:** Hes1 model parameters and definitions.

Symbol	Parameter Description	Nominal value	Parameter Range
*k*	degradation rate	0.03 min^−1^	fixed
*k*_1_	transport rate of protein	0.16*min* ^−1^	fixed
*P*_0_	regulatory transcription level of protein	2.4	0.24—24
*h*	Hill type coefficient	2	0.2—20
*ν*	rate of mRNA transcription	2.5 × 10^−2^ *min* ^−1^	2.5 × 10^−3^−2.5 × 10^−1^

The experimental design space is 𝔻, partially defined by an input *α* ∈ 𝕌 that modifies the transport rate, *k*_1_, as *α* × *k*_1_ where 𝕌 = [0.01,2] with a *δu* = 0.05. The input magnitude can be changed at admissible times, 𝓣 = {0,2,5,8,10,50,100,150,200} min. In this example, *u*_*b*_, is set to *α* = 1 ∀*τ* ∈ 𝓣. The allowable measurement state space includes the Hes1 mRNA, *m*, and the total protein concentration, *P*_1_+*P*_2_. The allowable sampling time space 𝕋 = [0, 300] min was defined to specify measurements with a *δt* = 10 min. We want the algorithm to specify a minimum of 8 distinct measurements. The experiment is designed to decrease the dynamical uncertainty on three target states, *m*, *P*_1_, and *P*_2_ under a target input *α* = 1 applied for the simulated time period.

To evaluate the MBDOE algorithm *in silico*, we simulate a plant model for which we want to reduce the dynamical uncertainty. The plant model is initially simulated with unknown model parameters set at the nominal values shown in Table 2. We generate 6 initial measurements of both mRNA, *m*, and total protein concentration, *P*_1_+*P*_2_, at times *t* = 10,20,60 min with 10% additive Gaussian noise.

**Fig 2:**
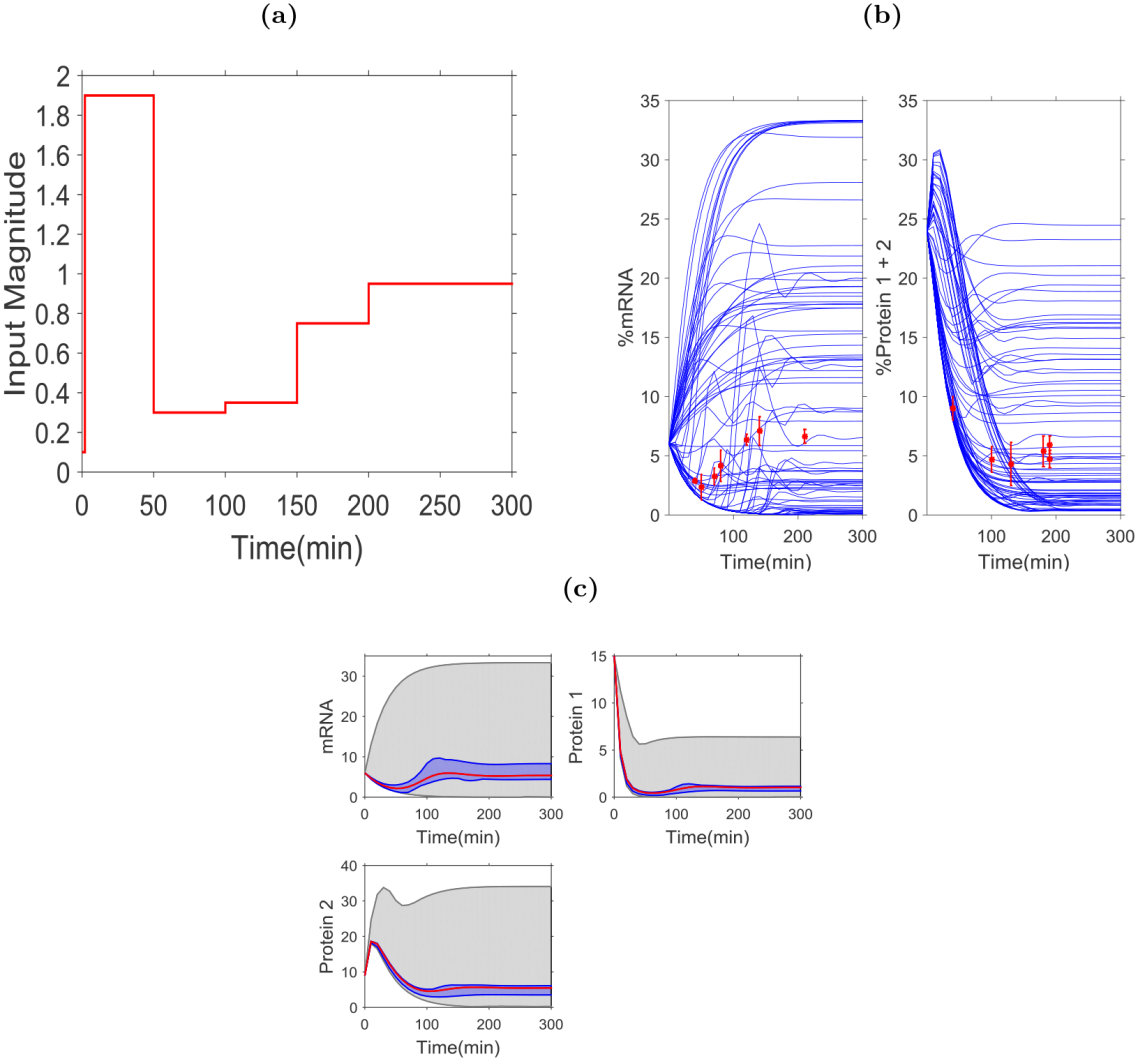
An illustration of the optimal experiment design for the Hes1 example. (**a**) The input sequence selected by the design for the duration of the experiment. (**b**) Model simulation of the output dynamics of the measurable species *m* and *P*_1_+*P*_2_ under an optimal input simulated with Ω_*RA*_. The red dots specify the selected optimal measurement points which are the mean values generated using nominal plant with additive 10% Gaussian noise. Error bars are also given by the standard deviation of the simulated data (three data points are generated for each time point). (**c**) The initial (grey) and final (blue) uncertainty in the target states dynamics. The designed experiment reduces the uncertainty region by 84%,81%, and 86% for mRNA, *P*_1_, and *P*_2_, respectively. The red line shows the simulated dynamics of the nominal plant.

Initial data identify the acceptable parameter space, Ω_*A*_, that is consistent with the data as defined by Eq (7). The resulting optimal experiment design is in Table 3. The optimal input sequence is specified by *α* values shown in Fig 2a and associated predicted measurements are shown on Fig 2b, superimposed on the representative dynamics for the measurable species to illustrate their connection with uncertainty. The greedy algorithm for the input selection is shown in Table 4 showing each iteration of input selection with the progressive predicted reduction in TDU values. The initial uncertainty and final uncertainty in the dynamics of the target states are superimpossed in Fig 2c to demonstrate the reduction of the region of uncertainty of the dynamics. The dynamical uncertainty regions associated with the mRNA, *P*_1_, *P*_2_ target states are reduced by 84%, 81% and 86%, respectively. In all cases, this reduced uncertainty region enclose the true dynamics of the system.

**Table 3:** Optimal Experiment Design for Hes1 Example.

Time Step	Action under MBDOE design	Scenario Tree Level
T = 0 mins	Apply *α* = 0.1	-
T = 2 mins	Apply *α* = 1.9	-
	Measure P1+P2	2^*nd*^
T = 40 mins	Measure mRNA	3^*rd*^
	Apply *α* = 0.3	-
T = 50 mins	Measure mRNA	3^*rd*^
T = 70 mins	Measure mRNA	3^*rd*^
T = 80 mins	Measure mRNA	3^*rd*^
	Apply *α* = 0.35	-
T = 100 mins	Measure P1+P2	3^*rd*^
T = 120 mins	Measure mRNA	1^*st*^
T = 130 mins	Measure P1+P2	2^*nd*^
T = 140 mins	Measure mRNA	3^*rd*^
T = 150 mins	Apply *α* = 0.75	-
T = 180 mins	Measure P1+P2	3^*rd*^
	Measure P1+P2	2^*nd*^
T = 190 mins	Measure P1+P2	3^*rd*^
T = 200 mins	Apply *α* = 0.95	-
T = 210 mins	Measure mRNA	3^*rd*^

**Table 4:** Greedy Method for Hes1 Input Vector Design.

Iteration	min(TDU)	τ*	u*	u at time 𝓣 = {0 2 5 8 10 50 100 150 200}
0	−	−	−	[1 1 1 1 1 1 1 1 1]
1	11.5191	50	0.3	[1 1 1 1 1 0.3 0.3 0.3 0.3]
2	10.7718	0	0.1	[0.1 0.1 0.1 0.1 0.1 0.1 0.3 0.3 0.3 0.3]
3	7.8597	2	1.9	[0.1 1.9 1.9 1.9 1.9 1.9 0.3 0.3 0.3 0.3]
4	7.6381	100	0.35	[0.1 1.9 1.9 1.9 1.9 1.9 0.3 0.35 0.35 0.35]
5	1.1810	150	0.75	[0.1 1.9 1.9 1.9 1.9 1.9 0.3 0.35 0.75 0.75]
6	0.5849	200	0.95	[0.1 1.9 1.9 1.9 1.9 1.9 0.3 0.35 0.75 0.95]

**Fig 3:**
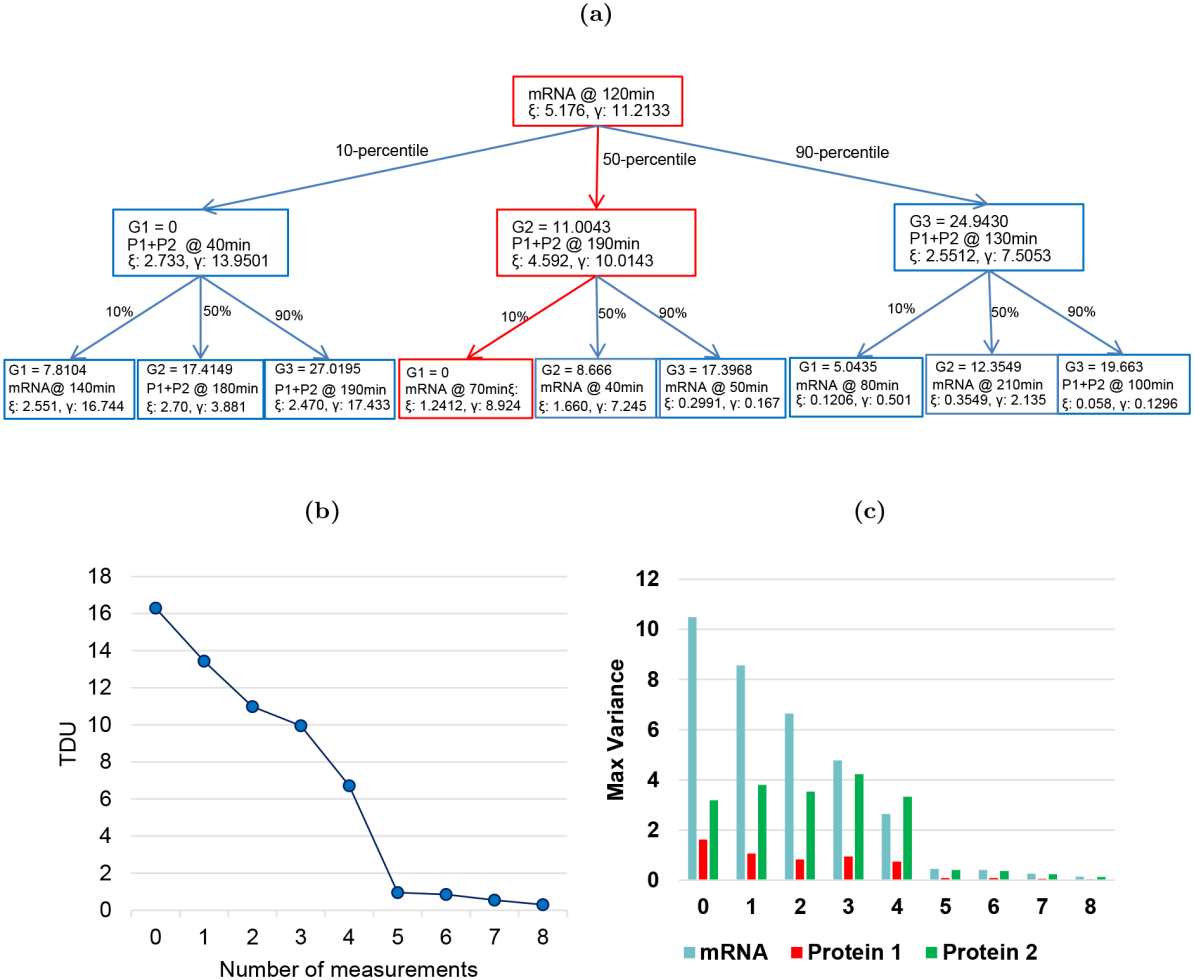
Supporting details of optimal experiment design for Hes1 example. (**a**) The measurement scenario tree representation for selecting optimal measurements. Each node defines, the measurement pairs with the predicted value of the distinguishability metric, *ξ*, target dynamical uncertainty, *γ*, and three estimated measurement values from simulation, G1, G2, and G3. The path along the scenario tree with predictions closest to the data from the nominal plant is shown in red. (**b**) Reduction of TDU and (**c**) individual variance of target states with each additional unique measurement from the scenario tree.

The measurement pairs are shown in the scenario tree in Fig 3a. the nodes of the tree specify the measurements that maximize uncertainty in the measurable species, *ξ*, while minimizing the TDU, *γ*, under the optimal input sequence, u*. In general, the values of *ξ* and *γ* decrease as you move down the tree and the predicted dynamics are constrained. To investigate how the measurement pairs contribute to the reduction of uncertainty in the dynamics of the target system, the total, *γ*, and individual variances are estimated assuming each predicted measurement has been taken (moving down and from left to right along the tree, neglecting repeated measurements). These results are shown in Fig 3b and 3c. These figures show that the first five unique measurements from the tree are necessary to resolve the target system dynamics.

To further explore the optimality of our MBDOE algorithm, we compare our results achieved with u* and M* to those of measurement only MBDOE. For this comparison, the measurement only MBDOE determined the optimal measurements, *M*_*T*_, assuming the applied input was the target input, **u**_**T**_. These results are summarized in Table 5 by the percentage reduction in the uncertain dynamical region for each target species and TDU. Our MBDOE algorithm outperforms all other experiment design combinations based on the TDU reduction. Although our MBDOE design is not the best in reducing the uncertain dynamical region for all target states, the results are comparable. The supplemental material contains the figures showing the uncertain dynamical regions for all measurement and input combinations in the Table. The supplemental material also contains figures that indicate the ability of our MBDOE algorithm to reduce the uncertain dynamical regions for different parameterizations of the Hes1 plant model.

**Table 5:** Comparison of Optimal Input MBDOE with Measurement Only MBDOE for the Hes1 Model.

Input Applied	Measurements	% Uncertainty Reduction			
		m	*P*_1_	*P*_2_	TDU, *γ*
u*	M*	84%	81%	86%	84.6%
**u**_**T**_	*M*_*T*_	75%	68%	81%	77%
u*	*M*_*T*_	86%	82%	83%	84.2%
**u**_**T**_	M*	80%	79%	82%	80.8%

The computational efficiency of this MBDOE method partially arises from the use of an interpolation as a surrogate of the model to reduce numerical integration of ODEs. The sparse grid terminated with a relative accuracy of 0.7% with an absolute tolerance of 0.9. The algorithm required only 137 model evaluations to build an interpolant which was sampled 10,000 times to identify 3852 acceptable parameters where *N*_*A*_ = 2000. The algorithm selected 48 representative parameters, *N*_*RA*_ = 48, to span the distinguishable dynamics generated by the acceptable parameters. For the input design, separate 1-D sparse grids are constructed for each admissible time of input change with 9 nodes. The total number model evaluation used for the complete experiment design is 18,716 where 2137 were for finding the representative parameters, 16,531 were used for the input selection, and 48 were used for the measurement selection. Clearly the majority of the model simulations were used to determine the optimal input signal. This is exceptionally large since we allowed the input to change at 9 time points and had fine input resolution with 40 different values. For comparison purposes, the Hes1 model experiment design in [16] required more than 30,000 model simulations to determine which species should be measured to provide the most information.

#### 19-Dimensional Example: The T-cell Receptor Model

The scalability of the MBDOE strategy to high dimensional problems is demonstrated using a T-cell receptor signaling model proposed by Lipniacki et al. [42]. This model was used previously as an example model to resolve dynamical uncertainty using parallel measurements only [15]. The model has 37 ODE equations that describes the dynamics of the species involved in the activation of T-cell signaling pathway, and 19 model parameters. Herein, we assume all of the parameters are unknown.

The Lipniacki model demonstrates the effectiveness of the MBDOE algorithm to use an optimal input to reduce dynamical uncertainty in a target system with conditions that maybe difficult to manipulate experimentally exactly. For illustrative purposes, we consider the system that could be experimentally determined to have a concentration of the agonist ligand, pMHC1, significantly higher than the concentration of the TCR receptors. The target system assumes that the concentration of this ligand pMHC1 predictably decreases as it binds to the TCR receptor. Two inputs are allowed to be manipulated in the experiment design under the system with constant supersaturating concentration of pMHC1: the drugs Sanguinarine, an Erk inhibitor, and PMA, an upstream activator of the protein kinase C. The magnitude levels of these control inputs, 𝕌, where 𝕌 ∈ [0, 1] with a resolution of *δu* = 0.3 can be changed at potential times 𝓣 = {5,10,15,20} min while the duration of an experiment run is 30 minutes. In this example, the initial u_*b*_ is set to no drug application. None of the drugs are applied to the target system.

The allowable measurement state space 𝕄 is defined to include phosphorylated Zap, pZAP, total phosphorylated Mek, pMEK + ppMEK, and total phosphorylated Erk, pERK + ppERK. The allowable sampling time space T = [0 30] min was defined to specify measurements with a *δt* = 1 min. Optimal measurements are designed under an optimal input to reduce dynamical uncertainty in target states, free SHP, ZAP, ppMEk, and ppERK under the target system with predictable decreasing levels of pMHC1. A total of four measurements are to be designed. The design is initiated by identifying acceptable parameters that fit an initial data set containing 9 points, pERK + ppERK at 0, 2, 5, 7, 8, 10, 12, 20 and 30 minutes.

**Fig 4:**
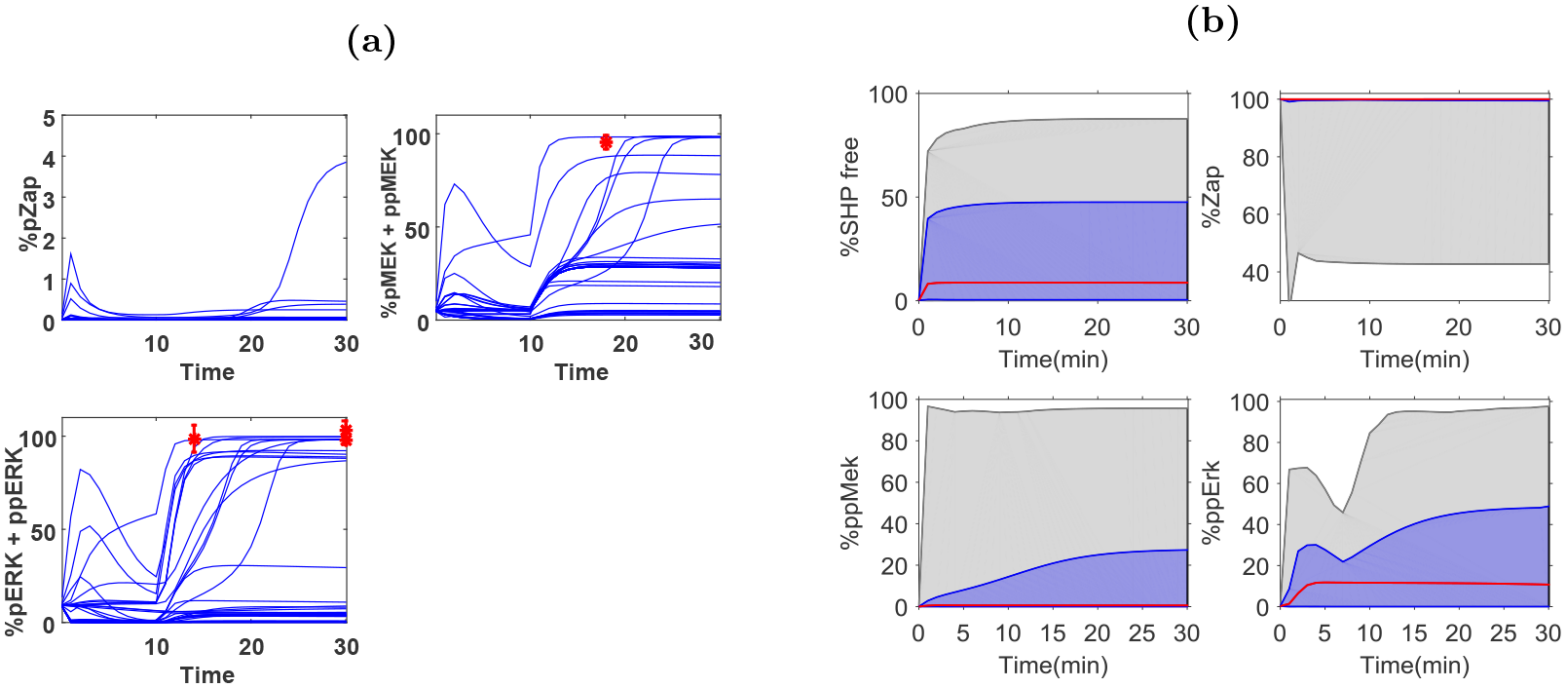
An illustration of the optimal experimental design for the TCR example. (**a**) Model simulation of the output dynamics of the measurable species under the optimal input provided in Table 6 simulated with Ω_*RA*_. The red dots specify the selected optimal measurement points by the mean values generated by nominal plant with additive 10% Gaussian noise. Error bars are also given by the standard deviation of the simulated data (three data points are generated for each time point). (**b**) The initial (grey) and final (blue) uncertainty in the target states dynamics. The designed experiment reduces the uncertainty region by 46%,99%,85% and 59% for free SHP, ZAP, ppMEK and ppERK, respectively. The red line shows the simulated dynamics of the nominal plant.

The optimal experimental design that constrains the predicted dynamics of the target system is presented in Table 6. Optimal drug levels for Sanguinarine and PMA are administered at times *t* = 10 minutes and optimal measurements are specified at times *t* = 14,18 and 30 minutes. These predicted measurements are also superimposed on the simulation of the representative parameters in Fig 4a to illustrate their connection with uncertainty. The optimal experimental design yields a reduction of uncertainty in target state dynamics by as much as 99% observed in Zap, with the least predicted dynamical uncertainty reduction by 46% for free SHP (Fig 4b). Although we would like to dramatically reduce the uncertainty for all target states, the reduction performance is highly dependent on the relationship between the target and measurable states. There are cases where the measurements may not be informative at all; our MBDOE approach will help inform the experimentalist *a priori*.

**Table 6:** Optimal Experiment Design for TCR Example.

Time Step	Action under MBDOE design	Scenario Tree Level
	Sanguinarine = 0	-
T = 10 mins	Apply PMA = 0.3	-
T = 14 mins	Measure pERK+ppERK	2^*nd*^
T = 18 mins	Measure pMEK+ppMEK	2^*nd*^
T = 30 mins	Measure pERK+ppERK	1^*st*^
T = 30 mins	Measure pERK+ppERK	2^*nd*^

**Fig 5:**
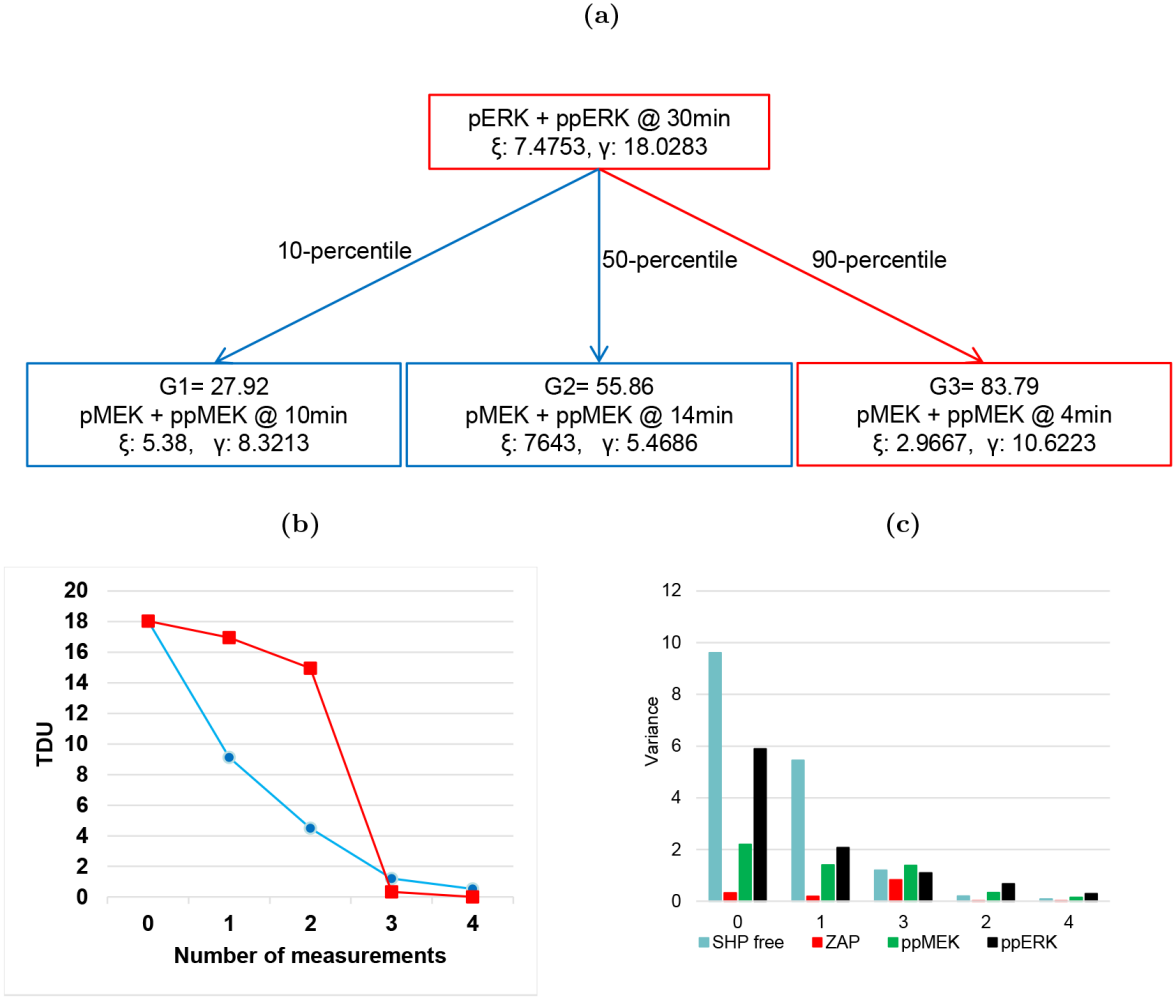
Supporting details of optimal experiment design for TCR example. (**a**) The measurement scenario tree representation for selecting optimal measurements. Each node defines, the measurement pairs with the predicted value of the distinguishability metric, *ξ*, target dynamical uncertainty, *γ*, and three estimated measurement values from simulation, G1, G2, and G3. The path along the scenario tree with predictions closest to the data from the nominal plant is shown in red. (**b**) Reduction of TDU (blue line) and (**c**) individual variance of target states with each additional unique measurement from the scenario tree. In (**b**) the reduction in TDU for a measurement only MBDOE scenario (red line) is provided for comparison purposes.

For completeness, the measurement scenario tree design is provided in Fig 5a. Each measurement from the scenario tree is investigated to determine how it contributes to the decrease in the TDU and the individual maximum variance (Fig 5b and 5c). Fig 5b also compares the reduction in TDU for our MBDOE approach to a measurement only design. For the measurement only MBDOE approach, we choose to find 4 measurements assuming the target input was applied to the TCR model. From this figure, it is clear that the optimization of the input signal is most important when a limited number of measurements are taken.

The computational efficiency of this MBDOE method on a higher dimension problem depends on the use of interpolation to explore the uncertain parameter space. From the 10,063 nodes used to create the grid, the algorithm identified 3974 acceptable parameters. The grid interpolant was sampled 100,000 times using LHS to identify an additional 403 acceptable parameters. The algorithm determined *N*_*RA*_ = 44 with *N*_*A*_ = 2000. There was no need to construct an input sparse grid since *N*_*u*_ = 16 and there were only 4 admissible times for potential input changes. The total number of model simulations for the complete experiment design is 16,991 with 12,063 for the selection of representative parameters, 4884 simulations for the input design, and 44 simulations to support the measurement selection. The majority of the model simulations are used to explore the large dimension uncertain parameter space to identify the acceptable and representative parameters. Given Eq (21) and the settings specified for the TCR model, the maximum number of model simulations possible was 19,084. The number of simulations actually performed was smaller than the worst case scenario because the input design terminated due to a lack of improvement in the TDU with additional changes to the input vector. For comparison purposes, previous work with this TCR model assuming different initial data and measurable species in [15] required 19,000 model evaluations to only define optimal measurements for a parallel design that fully resolved the dynamics to within experimental capabilities.

## Discussion

In this work, we have proposed an MBDOE algorithm that extends previous work to include the design of an optimal input together with optimal measurements to reduce dynamical uncertainty in biological systems. The global nature of the algorithm overcomes the challenges posed by traditional MBDOE designs that rely on the local FIM design. Furthermore, most MBDOE techniques that consider input design only compare a predefined set of input values and/or measurements. Our computationally efficient approach enables us to avoid this limitation and search over the input space for the optimal input values at pre-specified time points. Our strategy achieves a global design by implementing computationally efficient strategies using sparse grid interpolation, probability-weighted scenario trees, and dynamical representative parameters. An interpolant of the ODE system is created by strategically sampling the uncertain parameter space and is used to evaluate additional points by LHS without simulating the ODEs. Representative parameters are used to span the dynamics of the acceptable parameter space. Together, the representative parameters and the sparse grid interpolation are used to efficiently screen the dynamical variance on the input space and facilitate the probability-weighted scenario tree to identify associated optimal measurements that minimize the uncertainty in the target system dynamics. We have confirmed that our optimal input MBDOE algorithm will specify experiments that are more informative in their ability to reduce dynamical uncertainty over MBDOE techniques that only specify optimal measurements using 3D and 19D models. The optimization of the input signal is most important when a limited number of measurements are taken.

The computational expense of the MBDOE method is highly dependent on the dimension of the unknown parameter space and the feasible input space. For high dimension models, a large number of model simulations may be required to explore the large dimension uncertain parameter space to identify the acceptable and representative parameters. This computational burden can be reduced by the use of the sparse grid interpolation tool if the model output is smooth over the uncertain parameter space. For large input spaces, the majority of the model simulations in the MBDOE algorithm may be used to determine the optimal input signal. The number of model simulations increases with more flexibility in the input feasible space in terms of: (1) number of inputs that could be applied, (2) number of allowable changes in the inputs magnitudes (input resolutions), and (3) the number of admissible input times. In our examples, we specified a high degree of flexibility in the feasible input space for the lower dimension Hes1 model than the high dimension TCR model. As a result, the TCR model used 12,063 model simulations for the optimal input vector design while the Hes1 system required 16,531 model simulations.

Historically, MBDOE methods have designed experiments sequentially, whereby information gained from a previous experiment is used immediately to inform the next experiment design [32, 43, 44]. This approach is prevalent with the local MBDOE strategies since it improves the accuracy of the parameter estimates that support the design of the next experiment. The sequential design becomes problematic when the number of uncertain parameters is large because a single experiment does not estimate all the parameters accurately [45]. A parallel design methodology improves the estimates of the parameters and reduces experimental costs by specifying multiple measurements. The parallel design in [15] uses scenario trees to specify all optimal measurements necessary to resolve the experimentally distinguishable dynamics. A hybrid design combines the advantage of the sequential design with the cost efficiency of the parallel design by specifying multiple experiments to perform and iterating with the results informing the next round of MBDOE. Our MBDOE design uniquely supports this hybrid design strategy. It uses a measurement scenario tree to determine a user-specified number of measurements as opposed to the previous scenario tree approach that indicated all measurements required to fully resolve the dynamics. Herein, the number of desired measurements controls the depth of the constructed scenario tree. (If a tree is made too deep, we believe that many of the specified measurements would be sub-optimal since the actual dynamics only lie within a fraction of the predicted tree.) Thus, if the desired dynamical resolution is not achieved by one application of our MBDOE algorithm, the design can be repeated while informed with the new additional experimental data. New experimental data restricts the acceptable parameter space further to generate a new more relevant optimal experimental design.

Although our MBDOE algorithm is global, we cannot claim it is globally optimal. The algorithm makes optimal decisions within constraints. To make the problem tractable, we split the optimization problem into two sequential steps while the first specifies the optimal inputs and the second finds the associated measurement pairs. It is possible, that a better solution does exist and our algorithm misses it. Another source that may contribute to a sub-optimal result would be in the accuracy of the sparse grids. If the interpolants are not sufficiently accurate surrogates for the model responses, the results may be sub-optimal. In addition, we do not presume to have a good estimate of the number of acceptable parameter values needed to support the MBDOE since it is a function of the the biological system, the experimental setup, measurement noise, the mathematical model and its uncertainty. Hence, there is no exact formula for calculating the number of acceptable parameter vectors needed to support our experiment design algorithm. If the value is too small, the algorithm output would be sub-optimal and likely not repeatable. However, if repeated runs of our MBDOE algorithm produce similar results for increasing numbers of *N*_*A*_ than it is likely to be sufficiently large. Thus, without putting constraints on the biological system model, *N*_*A*_ must be sufficiently large to cover all distinguishable dynamics so that repeated application of our MBDOE algorithm derives similar optimal experiment designs.

Overall, the proposed MBDOE strategy successfully extends previous MBDOE capabilities to design an optimal input with associated measurements that minimizes the uncertainty in the target system dynamics. The interpolation grid provides a computational efficient way to search for both parameter fits and optimal input over large uncertain spaces. The use of representative parameters, selected to span the dynamical space of the biological model, provides a means to sample the dynamical space uniformly enhancing the computational tractability of this approach. Furthermore, the probability weighted scenario tree designs modified from [15] supports input and multiple optimal measurement pair selection with fewer sampled parameters. Although the enhancement of the discrimination ability of the experiment is somewhat model dependent, for the examples we have presented herein, we have found that the ability to specify an optimal input in addition to optimal measurements has enhanced the ability to bound the expected target system dynamics.

## Supporting Information

S1 Text Additional results to support the efficacy of the MBDOE algorithm to reduce dynamical uncertainty in model states. (PDF)
