# Supplementary material for: Efficient Optimization of Stimuli for Model-Based Design of Experiments to Resolve Dynamical Uncertainty
Source: PLoS Comput Biol. 2015 Sep 17;11(9):e1004488. doi: 10.1371/journal.pcbi.1004488 (PMC4574939; doi:10.1371/journal.pcbi.1004488)
Supplement: S1 Text — (PDF) [file pcbi.1004488.s001.pdf]

Efficient Optimization of Stimuli for Model-based Design of Experiments to Resolve Dynamical Uncertainty

Thembi Mdluli[1,*], Gregery T. Buzzard[2], Ann E. Rundell[1,*]
1 Weldon School of Biomedical Engineering, Purdue University, West Lafayette, IN, USA
2 Mathematics Department, Purdue University, West Lafayette, IN, USA
∗ E-mail: Corresponding; Thembi Mdluli: tmdluli@purdue.edu, Ann E. Rundell: rundell@purdue.edu

Supplementary Material

We demonstrate the importance of optimizing for the input by comparing the reduction in dynamical uncertainty for both the optimized input design and unoptimized input design and results are shown in Figure **S1**.

We further demonstrate that our algorithm is flexible and able to bound the dynamics of different plant systems. Using the Hes1 4-dimensional problem, four random parameter sets are selected within the range of parameter uncertainty to simulate the true system that generate data for the experiment design. These parameters are hidden to the user and the algorithm is allowed to select the optimal experiment design to discern each of the target dynamics. In Figure **S2**, we show the final dynamical uncertainty on the target states for each of the different randomly selected systems. This reduction in uncertainty bounds the true dynamics of each of the true system.

(a)

Time Step	Action under MBDOE Design	Number of Scenario Tree Level
T = 0 mins	Apply α = 1	
T = 40 mins	Measure P1+P2<br>Measure mRNA	2nd<br>3rd
T = 90 mins	Measure mRNA	3rd
T = 110 mins	Measure mRNA<br>Measure mRNA	1st<br>3rd
T = 130 mins	Measure P1+P2	2nd
T = 140 mins	Measure mRNA	3rd
T = 180 mins	Measure P1+P2<br>Measure P1+P2	2nd<br>3rd
T = 190 mins	Measure P1+P2	3rd
T = 200 mins	Measure P1+P2	3rd

(b)

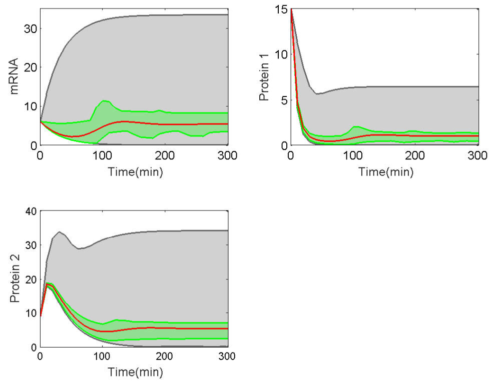

(c) **(d)**

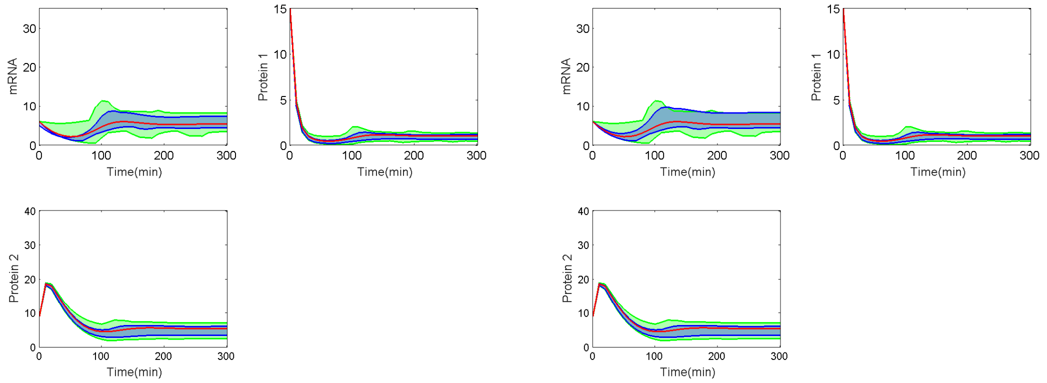

Figure S1. Comparison of Optimal Input MBDOE with Measurement Only MBDOE. (**a**) Optimal measurement pairs for the experimental design with only target input applied. (**b**) The resolution of the target states dynamics for the unperturbed system with target input $\mathbf{u}_T$ and corresponding optimal measurements M_T. Grey shade shows the original uncertainty region in the states and the green shade shows the final uncertainty region with measurement only optimal design. The green shade shows a percentage decrease of the uncertain region from the grey region that correspond to $75\%, 68\%$ and 81% for mRNA, P_1 and P_2, respectively.(**c**) Resolution of target states dynamics with target input $\mathbf{u}_T$ (green shade) and under MBDOE optimal input $\mathbf{u}^*$ (blue shade) given optimal measurements selected through the measurement only design M_T. The blue shaded uncertainty region shows a percentage decrease of the uncertain region corresponding to $86\%, 82\%$ and 83% while the green shade shows a decrease corresponding to $84\%, 79\%$ and 68% for mRNA, Protein1 and Protein 2, respectively. (**d**) Resolution of target states dynamics with target input $\mathbf{u}_T$ (green shade) and with MBDOE optimal input $\mathbf{u}^*$ (blue shade) given the optimal measurements associated with the optimal input design, M^*. The blue shade shows a percentage decrease of the uncertain region corresponding to $84\%, 81\%$, and 86% while the green shade shows a decrease corresponding to $80\%, 79\%$, and 82% for mRNA, Protein1 and Protein 2, respectively. In all the cases, the optimal input design gives the overall best resolution in the dynamics of the target system.

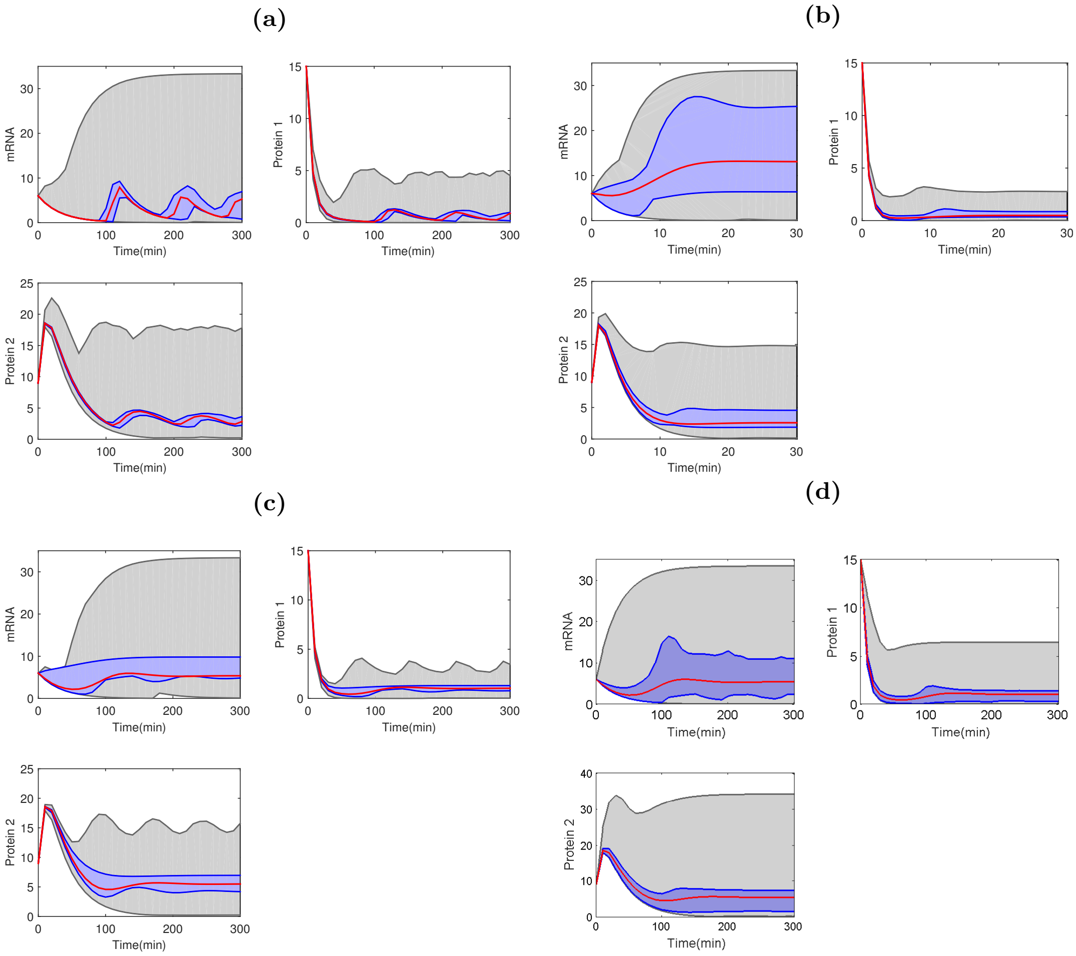

Figure S2. Reduction of uncertainty for systems generated with a hidden randomly selected true parameter set. The lowest worst case percentage reduction in the uncertainty regions for each of the cases: (**a**) is 78% observed in mRNA, (**b**) is 34% observed in mRNA, (**c**) is 74% observed in mRA and (**d**) is 52% observed in mRNA. The parameter sets used for each of the plants are for unknown parameter set, $(P_0, h, \nu)^*$: (**a**) $(2.4, 20, 0.025)$, (**b**) $(1.3637, 0.6870, 0.0048)$, (**c**) $(2, 2, 0.25)$, (**d**) $(1.4, 1.12, 2 \times 10^{-2})$
